# Soft Tissue Reconstruction Does Not Compromise Infection Control in Chronic Knee Periprosthetic Joint Infection Treated with Two-Stage Exchange Arthroplasty Despite Increasing Complexity

**DOI:** 10.3390/microorganisms14030682

**Published:** 2026-03-18

**Authors:** Carlos Mendoza Aguiló, Matías Vicente, Antonio Cano, José Antonio López Martínez, Antonio Bulla, Carles Amat, Jordi Serracanta, Pablo S. Corona

**Affiliations:** 1 Orthopaedic Surgery Department, Vall d’Hebron University Hospital, Universitat Autònoma de Barcelona (UAB), 08035 Barcelona, Spain; matias.vicente@vallhebron.cat (M.V.); carles.amat@vallhebron.cat (C.A.); pablosalvador.corona@vallhebron.cat (P.S.C.); 2 Department of Surgery, Universitat Autònoma de Barcelona (UAB), 08035 Barcelona, Spain; 3 Septic and Reconstructive Surgery Unit (UCSO), Orthopaedic Surgery Department, Vall d’Hebron University Hospital, 08035 Barcelona, Spain; antonio.cano@vhir.org; 4 Musculoskeletal Tissue Engineering Group, Vall d’Hebron Institute Research, Vall d’Hebron University Hospital, 08035 Barcelona, Spain; 5 Department of Plastic Surgery and Major Burn, Vall d’Hebron University Hospital, Universitat Autònoma de Barcelona (UAB), 08035 Barcelona, Spain; joseantonio.lopez@vallhebron.cat (J.A.L.M.); antonio.bulla@vallhebron.cat (A.B.); jordi.serracanta@vallhebron.cat (J.S.)

**Keywords:** knee, periprosthetic joint infection, two-stage exchange, soft tissue reconstruction, medial gastrocnemius flap, microsurgical reconstruction, anterolateral thigh free flap, orthoplastic surgery

## Abstract

The role of soft tissue reconstruction in infection control of knee periprosthetic joint infection (PJI) treated with a two-stage exchange strategy remains controversial. This retrospective observational study analysed consecutive patients with chronic knee PJI managed with a two-stage protocol between 2010 and 2023, comparing outcomes between cases requiring flap-based soft tissue reconstruction and those achieving primary closure. A total of 118 patients with a minimum follow-up of 24 months were included. Forty patients (33.9%) required soft tissue reconstruction (STR), including 25 pedicled medial gastrocnemius flaps and 15 anterolateral thigh (ALT) microsurgical free flaps. Patients requiring STR showed greater baseline complexity, with a higher number of previous surgical procedures (3.03 vs. 2.08; *p* = 0.0057) and a higher prevalence of diabetes mellitus and sinus tracts. Despite this, infection control was superior compared with non-reconstructed cases (100% vs. 88.5%; *p* = 0.029). Within the STR group, both pedicled and microsurgical techniques achieved complete infection eradication; however, ALT flaps were associated with higher complication rates (46.7%), with partial flap necrosis being the most frequent event. The use of microsurgical reconstruction increased progressively over time, reflecting growing reconstructive complexity. Formal soft tissue reconstruction does not compromise and may facilitate infection control within a multidisciplinary pathway.

## 1. Introduction

Total knee arthroplasty is one of the most effective orthopaedic procedures for advanced osteoarthritis [1]; however, periprosthetic joint infection (PJI) remains one of its most serious and difficult-to-treat complications [2]. Although single-stage revision may be appropriate in selected cases, two-stage replacement arthroplasty remains the most widely accepted treatment strategy worldwide for chronic knee PJI [3,4].

Regardless of the revision protocol, the condition of the periarticular soft tissues plays a pivotal role in infection management. The infection itself and repeated surgical interventions can compromise the soft tissue envelope, resulting in fistulas, retracted scarring, necrosis, wound dehiscence, or established coverage defects that prevent primary closure [5]. Such alterations may jeopardise infection eradication, promote implant failure, and ultimately lead to knee arthrodesis or above-the-knee amputation [6].

Several reconstructive options are available, ranging from pedicled to free microvascular flaps [7]. The medial gastrocnemius flap is the most commonly employed pedicled option, owing to its simplicity, anatomical proximity, reliable vascularity, and adequate tissue volume [8]. Nevertheless, extensive defects, proximal exposure, or severe soft tissue compromise may require free microvascular reconstruction, allowing for tailored coverage according to defect size and tissue requirements [7,8,9].

Despite the clinical relevance of soft tissue reconstruction (STR) in knee PJI, comparative evidence evaluating infection outcomes in patients managed with and without formal STR remains scarce. Furthermore, few studies have analysed outcomes according to the complexity of the reconstructive strategy employed. Therefore, this study was designed to test two hypotheses: first, that patients requiring formal STR would not demonstrate inferior infection control compared with those managed without STR; and second, that reconstructive complexity has increased over time, reflected by a growing need for microvascular free flaps. Accordingly, the primary aim was to evaluate infection control in chronic knee PJI patients treated with and without STR, and, within the reconstruction group, to compare outcomes between pedicled medial gastrocnemius flaps and free microvascular flaps. A secondary objective was to analyse temporal trends in reconstructive complexity.

## 2. Materials and Methods

### 2.1. Study Design and Patient Selection

Following institutional review board approval (IRB-PR(AT)504/2025), a retrospective observational study was conducted including all consecutive patients treated for chronic knee periprosthetic joint infection using a two-stage exchange protocol at a 1000-bed tertiary university hospital between 2010 and 2023. All procedures were performed within a dedicated multidisciplinary Septic Unit at a nationally recognised referral centre for musculoskeletal infection.

Inclusion criteria were: (1) a confirmed diagnosis of chronic PJI according to the European Bone and Joint Infection Society (EBJIS) definition [10]; (2) treatment with a two-stage arthroplasty replacement strategy; and (3) a minimum follow-up of 24 months after reimplantation. Patients with shorter follow-up or who did not meet all inclusion criteria were excluded. Demographic, clinical, surgical, and microbiological data were collected retrospectively from electronic medical records. When necessary, missing information was obtained through structured telephone interviews with patients or their relatives.

### 2.2. Study Groups

Patients were stratified into two groups according to the requirement for soft tissue reconstruction:

- **No Soft Tissue Reconstruction (N-STR) group:** Patients treated with a standard two-stage revision procedure without formal flap coverage.
- **Soft Tissue Reconstruction (STR) group:** Patients requiring additional reconstructive procedures to address soft tissue deficiencies, including medial gastrocnemius pedicled flaps or free microvascular flaps.

### 2.3. Outcome Measures and Definitions

The primary endpoint was infection control. Host-related variables included diabetes mellitus, malignancy, immunosuppression, American Society of Anesthesiologists (ASA) score, and Charlson comorbidity index. Surgical variables included the dates of surgery, type of spacer (articulated or static), isolated microorganisms, and characteristics of the final prosthesis.

Treatment success was defined according to the Delphi consensus criteria proposed by Diaz-Ledezma et al. [11], which required: (a) a healed wound without sinus tract, persistent drainage, or pain and without recurrence caused by the same microorganism; (b) no reoperation for infection after reimplantation; and (c) no PJI-related mortality. Failure was further defined as the need for lifelong suppressive antibiotic treatment or the development of a new PJI caused by a different microorganism.

### 2.4. Operative Technique Description

All procedures were performed by one of four senior surgeons from the dedicated Infection Unit following a standardised two-stage protocol. Differences between groups were limited to the indication, technique, and timing of soft tissue reconstruction.

During the first stage, a medial parapatellar approach was used and extended through a tibial tubercle osteotomy when safe mobilisation of the extensor mechanism or adequate exposure could not be achieved. The prosthesis and cement were removed, followed by aggressive debridement. At least six tissue samples were obtained for microbiological culture before antibiotic administration. The joint was irrigated with saline without additives using a low-pressure system, and an antibiotic-loaded cement spacer (Vancogenx^®^, Tecres, Sommacampagna, Italy) was implanted using vancomycin–gentamicin-loaded acrylic bone cement (Vancogenx^®^, Tecres SpA, Italy), supplemented with an additional 4 g of vancomycin and 4 g of tobramycin per 40 g cement bag. Static spacers were preferred in cases of major soft tissue deficiency, extensor mechanism compromise, or extensive bone loss. The distribution of spacer type differed between groups according to local soft tissue conditions. In the soft tissue reconstruction (STR) group, all patients (100%) received a static spacer. In the no soft tissue reconstruction (N-STR) group, 61 patients received an articulated spacer and 17 received a static spacer. Antimicrobial management was conducted within our multidisciplinary Infection Unit according to a structured institutional protocol and individualized on the basis of intraoperative microbiological findings. Empirical intravenous therapy was initiated only after adequate sampling had been obtained and was subsequently adjusted according to culture results and antimicrobial susceptibility testing. Intravenous treatment was typically maintained for approximately 8–10 days. In most cases, a beta-lactam agent constituted the first-line empirical regimen. When resistant organisms were suspected or confirmed, broader antimicrobial coverage was implemented, including carbapenem-based regimens and, when indicated, agents active against resistant Gram-positive pathogens. Following clinical stabilization and once definitive microbiological results were available, patients were transitioned to oral antibiotic therapy, which was continued for at least six weeks. In cases of Gram-positive infection, combination therapy including rifampicin was preferentially employed due to its biofilm activity, particularly in staphylococcal infections. For susceptible Gram-negative organisms, fluoroquinolones were used when appropriate. Reimplantation was undertaken only after clinical resolution of infection had been achieved. In addition to the absence of local inflammatory signs, criteria included adequate nutritional and metabolic optimization and a consistent downward trend in inflammatory markers, particularly C-reactive protein. Consequently, the interval between stages was determined by patient-specific clinical and laboratory parameters rather than a predefined chronological schedule.

**Figure 1:**
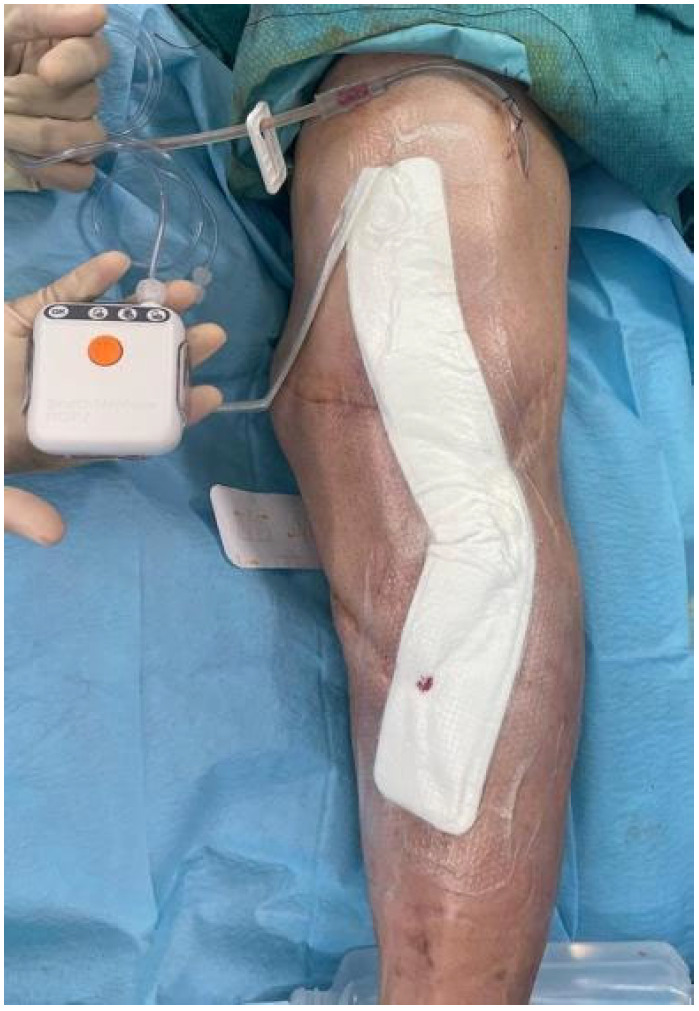
Intraoperative view of prophylactic incisional negative pressure wound therapy (iNPWT; PICO^®^, Smith & Nephew, Memphis, TN, USA) applied as part of standard wound management during revision surgery for infected total knee arthroplasty.

During the second stage, the spacer was removed, repeat debridement and sampling were performed, and definitive reconstruction was undertaken. Implant selection was based on bone loss, ligamentous stability, and bone quality; in most cases, a cemented modular rotating-hinge prosthesis was used (Endo-Model^®^-M; Waldemar Link, Hamburg, Germany). Prophylactic incisional negative pressure wound therapy (PICO^®^, Smith & Nephew; Memphis, TN, USA) was frequently applied for a minimum of 14 days in the vast majority of patients in both stages of treatment (Figure 1).

### 2.5. Soft Tissue Reconstruction Strategies

**Figure 2:**
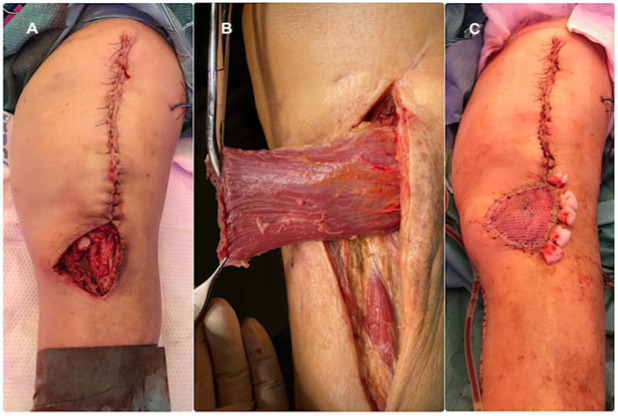
(**A**) Moderate infrapatellar soft tissue defect in an infected total knee arthroplasty precluding primary closure, amenable to coverage with a medial gastrocnemius flap. (**B**) Medial gastrocnemius flap after elevation, demonstrating its limited dimensions and restricted arc of rotation. (**C**) Final appearance after split-thickness skin grafting.

**Figure 3:**
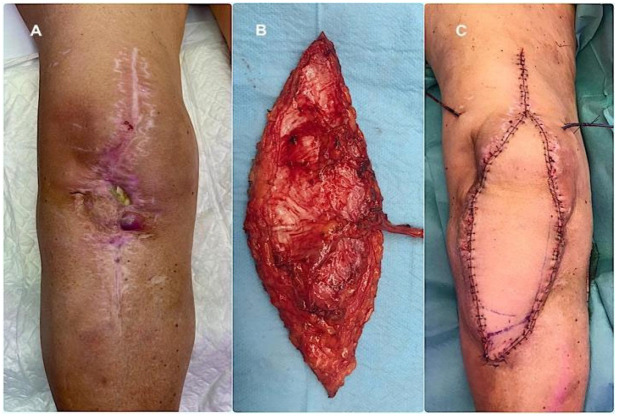
(**A**) Complex soft tissue defect in an infected total knee arthroplasty precluding coverage with a medial gastrocnemius flap. (**B**) Anterolateral thigh (ALT) free flap after harvest, demonstrating its long vascular pedicle. (**C**) Final appearance after comprehensive soft tissue reconstruction, including the defect and surrounding scarred tissues.

Soft tissue reconstruction was planned and executed using a multidisciplinary orthopaedic–plastic surgery approach within the dedicated Infection Unit. When feasible, medial gastrocnemius pedicled flaps were preferentially performed during the second stage of the two-stage revision protocol (Figure 2). A comprehensive three-stage protocol was developed by our unit [12] and adopted for patients requiring microsurgical reconstruction, with the anterolateral thigh (ALT) flap used as the preferred free flap (Figure 3). The rationale for selecting a fasciocutaneous flap, particularly for anterior knee defects, was based on several considerations. First, fasciocutaneous tissue provides a more like-to-like reconstruction in an area subjected to repetitive flexion–extension movements and increased risk of friction, erosion, and superficial trauma. Second, fasciocutaneous flaps facilitate safer and easier re-elevation during subsequent surgical stages, particularly at the time of definitive joint reconstruction, compared with musculocutaneous alternatives. Under this three-stage protocol, surgical management is temporally divided in three discrete treatment phases; the first phase consisted of standard prosthesis removal, aggressive debridement, static spacer implantation, and temporary wound management, most commonly using negative pressure wound therapy. Definitive soft tissue reconstruction was performed during the second phase, ideally within five days, following preoperative CT angiography to assess vascular anatomy and plan microsurgical anastomoses. Definitive joint reconstruction phase was subsequently undertaken once infection control had been confirmed, the flap demonstrated stable integration, and the patient had achieved adequate physiological optimisation, following the principles of a conventional second-stage reimplantation.

Immediate reconstruction was selectively performed in two specific scenarios: during the second stage when a planned medial gastrocnemius flap was indicated, following prosthesis implantation; or during the first stage in cases of acute soft tissue loss with spacer exposure, provided that coverage with a medial gastrocnemius flap was feasible. In these cases, the flap was re-elevated during the reimplantation procedure to allow spacer removal and definitive prosthetic reconstruction.

### 2.6. Statistical Analysis

Statistical analyses were performed using GraphPad Prism^®^ version 9.0. Categorical variables were compared using Fisher’s exact test, and continuous variables were compared using Student’s *t*-test or Mann–Whitney U test, depending on the distribution of the data. Normality was assessed using the Shapiro–Wilk test. All tests were two-tailed, and a *p*-value < 0.05 was considered statistically significant. Results are presented with the corresponding 95% confidence intervals. Exact 95% confidence intervals for proportions were calculated using the Clopper–Pearson method.

## 3. Results

A total of 118 patients treated for periprosthetic knee joint infection (PJI) were included in the study. The mean age of the cohort was 69.7 ± 10.2 years, and comorbidities were common, as was a history of multiple previous surgical interventions. Baseline demographic and clinical characteristics are detailed in Table 1.

Of the 118 patients, 78 (66.1%) were treated without formal soft tissue reconstruction (N-STR group), while 40 (33.9%) required additional soft tissue coverage (STR group). The gender distribution was similar between the groups (N-STR: 44.9% men; STR: 46.5% men), and patients in the STR group were slightly younger on average (68.0 ± 12.45 vs. 70.5 ± 9.27 years), although this difference was not statistically significant (*p* = 0.4206).

In terms of comorbidities and infection-related risk factors, no statistically significant differences were observed between groups. Diabetes mellitus (39.5% vs. 28.2%, *p* = 0.2887), the presence of a sinus tract (44.8% vs. 26.9%, *p* = 0.1022), and malignancy (14.1% vs. 2.6%, *p* = 0.1002) showed numerical differences without reaching statistical significance. Similarly, no significant differences were found in smoking status, obesity, Charlson comorbidity index, alcohol consumption, inflammatory diseases, cirrhosis, anticoagulant treatment, intra-articular pus, or suppressive antibiotic therapy.

Microbiological findings were comparable between groups, with no statistically significant differences in pathogen distribution (Table 2). *Methicillin-sensitive Staphylococcus aureus* (MSSA) was isolated in 12.5% of patients in the STR group and 3.85% in the N-STR group (*p* = 0.1903), and *Enterococcus faecalis* was identified in 12.5% and 3.85% of cases, respectively (*p* = 0.1903). *Enterobacter* spp. was isolated more frequently among patients who required soft tissue reconstruction (9.38% vs. 1.28%), which was close to statistical significance (*p* = 0.0732), while the other microorganisms were detected in low and comparable proportions. Regarding polymicrobial infections, these were observed in 21.42% of patients in the STR group and 15.68% in the N-STR group (*p* = 0.690).

Patients in the STR group had undergone significantly more prior surgical procedures than those in the N-STR group (3.03 ± 1.96 vs. 2.08 ± 1.32; *p* = 0.0057), whereas the distribution of ASA scores did not differ significantly between the two groups (*p* = 0.8896). Spacer type distribution differed significantly between groups. All patients in the STR group (100%) were treated with a static spacer, whereas in the N-STR group 61 patients (78.2%) received an articulated spacer and 17 (21.8%) a static spacer.

With a minimum follow-up of 24 months (range: 24–120 months), the overall infection control rate after two-stage replacement arthroplasty was 92.4% (95% CI: 86.0–92.4%). The absence of infection at final follow-up was achieved in 88.46% of patients treated without soft tissue reconstruction (N-STR) (95% CI: 79.2–94.6%) and in 100% of those requiring soft tissue reconstruction (STR) (95% CI: 91.2–100%), representing a statistically significant difference (*p* = 0.0294).

Within the soft tissue reconstruction cohort, 25 patients underwent medial gastrocnemius (MG) flap reconstruction and 15 patients received an anterolateral thigh (ALT) free flap. The distribution by age and sex was comparable between the groups (Table 3). Diabetes mellitus was significantly more prevalent among patients with GM (60.87% vs. 6.67%; *p* = 0.0017), while inflammatory diseases (26.67% vs. 0%; *p* = 0.0185) and immunodeficiency (53.33% vs. 0%; *p* = 0.0001) were significantly more common in the ALT group. Intra-articular pus at the time of surgery was also observed more frequently in ALT patients (71.43% vs. 8.33%; *p* = 0.0095), while no significant differences were found in the Charlson comorbidity index, smoking, or obesity. Although the number of previous surgical interventions tended to be higher in the GM group (3.3 ± 1.65 vs. 2.67 ± 2.24; *p* = 0.1335), ALT patients required a significantly higher number of subsequent surgical procedures (2.5 ± 0.54 vs. 0.75 ± 0.31; *p* = 0.0022).

Microbiological profiles did not differ significantly between GM and ALT reconstructions (Table 4). The rate of positive cultures, defined as those obtained intraoperatively during the STR, was 32% in the GM group and 40% in the ALT group, (*p* = 0.74). Regarding polymicrobial infections, these were observed in 25% of patients in the GM group and 16.7% in the ALT group (*p* = 0.999). The distribution of pathogen types was comparable between groups.

Postoperative complications were significantly more frequent in the ALT group (*p* = 0.0049). In the GM group, 94.1% of patients experienced no complications (95% CI: 73.0–99.7%), whereas in the ALT group only 46.7% were complication-free (95% CI: 21.3–73.4%). The overall complication rate in the ALT group was 53.3% (95% CI: 26.6–78.7%). Partial flap necrosis occurred in 26.7% of ALT cases (95% CI: 7.8–55.1%), and total flap necrosis in 13.3% of cases (95% CI: 1.7–40.5%) (Table 5). Despite these differences, infection control was achieved at final follow-up in 100% of patients in both reconstruction subgroups.

**Figure 4:**
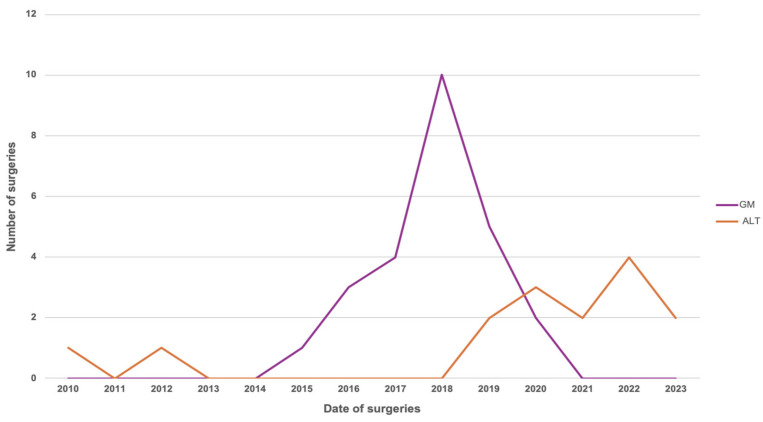
Time trend analysis (2010–2023) showing the evolution in the use of medial gastrocnemius and anterolateral thigh (ALT) flaps for soft tissue reconstruction.

Finally, analysis of temporal trends showed a progressive change in the distribution and complexity of soft tissue reconstruction strategies over the last 13 years of the study period (Figure 4).

During the early years, pedicled medial gastrocnemius flaps accounted for the majority of reconstructive procedures, while free microvascular flaps were used sporadically. Starting in 2018, there was a sustained annual increase in the number and proportion of anterolateral thigh free flaps, which equalled and subsequently surpassed medial gastrocnemius flaps in frequency starting in 2020.

## 4. Discussion

In this cohort of chronic knee periprosthetic joint infections treated with a standardised two-stage exchange protocol, approximately one-third of patients required formal soft tissue reconstruction, reflecting the high clinical and biological complexity of contemporary PJI management. Consistent with our primary hypothesis, the need for soft tissue reconstruction was not associated with inferior infection control. On the contrary, despite a significantly higher burden of previous surgeries and more advanced infection severity, patients requiring STR achieved superior infection control compared with those managed without STR (100% vs. 88.5%, *p* = 0.0294). These findings indicate that, when appropriately integrated into a structured orthoplastic treatment pathway, soft tissue reconstruction may counterbalance—rather than exacerbate—the negative prognostic impact of severe local compromise.

This observation contrasts with the findings reported by Baltzer et al., who analysed a cohort of 218 knee periprosthetic joint infections, 83.5% of which were managed using a two-stage strategy [13]. In that study, the requirement for soft tissue reconstruction—together with increased surgical complexity—was identified as a factor associated with a higher risk of infectious failure in a population treated with mixed one- and two-stage revision strategies. Specifically, patients requiring flap coverage demonstrated a significantly lower likelihood of complete healing (30.8% vs. 66.2%, *p* = 0.001) and a higher need for suppressive antibiotic therapy (42.3% vs. 15.6%, *p* = 0.003) compared with those not requiring a flap. In contrast, our study did not demonstrate a negative impact of soft tissue reconstruction on infection control. Similarly, Moog et al. reported in a retrospective cohort of 78 patients with knee PJI that infection-free survival was achieved in 83% of patients undergoing soft tissue reconstruction compared with 57% of those managed without reconstruction (*p* = 0.0376) [14]. These findings support the concept that restoration of a stable, well-vascularized soft tissue envelope facilitates infection eradication by improving local perfusion and antibiotic delivery while reducing dead space [15].

Early and fully integrated collaboration between orthopaedic and plastic surgery teams represents a cornerstone in the management of complex knee periprosthetic joint infection and is a defining feature of our institutional model. In this context, Colen et al. [16] showed that patients managed within a coordinated multidisciplinary framework with early plastic surgery involvement achieved higher prosthesis salvage rates and a lower risk of amputation compared with those referred after multiple prior interventions, reporting an overall infection control rate of 61.6%. Importantly, they identified the number of previous knee operations prior to definitive soft tissue reconstruction as an independent predictor of reduced prosthesis salvage and increased limb loss. Consistent with these observations, patients requiring soft tissue reconstruction in our series exhibited a markedly more compromised clinical profile. They had undergone a significantly higher number of prior surgical procedures (3.03 vs. 2.08 ± 0.15; *p* = 0.0057) and showed higher prevalence of sinus tract formation (44.8% vs. 26.9%; *p* = 0.102) and diabetes mellitus (39.5% vs. 28.2%; *p* = 0.288), factors classically associated with impaired wound healing and reduced soft tissue resilience, but without reaching statistical significance. Similarly, Christiner et al. [17] reported that two or more major prior operations significantly increase failure rates following two-stage revision, highlighting the cumulative detrimental effect of repeated surgical trauma. Taken together, these findings indicate that patients ultimately requiring soft tissue reconstruction represent a subgroup in whom adverse biological and surgical risk factors accumulate, reflecting progressive soft tissue deterioration, compromised local vascularity, and diminished healing capacity. This is further supported by the findings of Song et al. [18] who reported inferior infection-free survival (80% vs. 94%, *p* = 0.006) and lower microorganism identification rates (69% vs. 88%, *p* = 0.006) in patients undergoing surgical intervention prior to referral to a specialised PJI centre. Collectively, these data underscore the importance of early referral and centralisation of complex PJI management in specialised, high-volume centres, in order to minimise unnecessary prior procedures and prevent progressive compromise of the soft tissue envelope [19].

Within the reconstruction subgroup, pedicled medial gastrocnemius (MG) flaps and free anterolateral thigh (ALT) flaps reflected fundamentally different clinical scenarios rather than interchangeable reconstructive options. Pedicled gastrocnemius flaps provided reliable coverage for small-to-moderate anterior and infrapatellar defects, as supported by Houdek et al., who reported 10-year revision-free and amputation-free survival rates of 68% and 79%, respectively. Importantly, wound size ≥ 50 cm^2^ was identified as a significant predictor of reconstructive failure, supporting the limitation of this technique to moderate-sized defects [20]. In contrast, free microvascular flaps were predominantly required in patients with extensive soft tissue loss and advanced biological compromise, including intra-articular purulence, immunodeficiency, and inflammatory comorbidities. These patients required more surgical procedures and experienced higher complication rates, reflecting a more complex host and local environment. Consistent with our findings, Lee et al. reported limb salvage in 82% of patients treated with free flaps, although 18% ultimately required amputation and functional outcomes remained limited, while Hamrouni et al. achieved limb salvage in all cases at the cost of a substantial early complication rate [21,22]. Taken together, these data indicate that, while free tissue transfer is superior for the management of large or complex soft tissue defects, it identifies a distinct and more compromised patient population and is associated with greater surgical burden and potential functional limitations. Moreover, its implementation requires specialised expertise and is not universally available. Therefore, pedicled flaps should be reserved for localised, moderate defects, whereas microvascular reconstruction should be considered for extensive soft tissue loss within specialised, multidisciplinary centres.

An interesting finding regarding microbiological profiles between reconstructive groups was that the use of temporary negative pressure wound therapy (NPWT) in the anterolateral thigh (ALT) free flap group was not associated with a higher rate of polymicrobial positive cultures. Previous studies have linked temporary NPWT to an increased incidence of polymicrobial infection and a higher risk of multidrug-resistant organisms in orthopaedic and trauma populations. Valenzuela et al. [23] reported that open wound management, including NPWT, can convert monomicrobial to polymicrobial periprosthetic joint infection in 34% of cases, with only 45% of patients who converted to polymicrobial infection remaining infection-free at follow-up, compared with 70% of those who remained monomicrobial. In contrast, no such difference was observed in our cohort, as we observed polymicrobial cultures in only 16.7% of patients in the ALT group, with a subsequent infection eradication rate of 100%. This finding may be explained by the implementation of a strict and standardized wound management protocol. In cases managed using a three-stage reconstruction strategy, NPWT was applied for a maximum of five days, during which the wound remained sealed and isolated from the external environment. This limited exposure, combined with the local antibacterial effect of the antibiotic-loaded spacer, may mitigate the increased microbiological risk associated with prolonged NPWT use. Based on these observations, NPWT should be restricted to short, predefined intervals and employed only when definitive flap reconstruction is already scheduled.

A particularly relevant finding of this study is the progressive and sustained increase in the use of microvascular free flaps throughout the study period—a trend that has become especially pronounced in recent years. The contemporary literature from high-volume centres consistently describes a growing reliance on microvascular reconstruction as a limb salvage strategy in infected prosthetic knees with complex soft tissue defects, particularly in scenarios in which pedicled options are insufficient to provide stable and durable coverage [24]. This reconstructive transition appears to reflect a substantial transformation in the clinical and biological profile of patients referred to tertiary centres, characterized by greater cumulative surgical burden, more advanced host compromise, and more extensive and complex soft tissue deficiencies, rather than a simple shift in reconstructive team preference or technical approach. This evolving landscape is likely to define the future of limb salvage in complex periprosthetic knee infections, underscoring the need for reconstructive teams to be prepared for an increasing reliance on advanced microvascular techniques and multidisciplinary, protocol-driven care.

We acknowledge the limitations of our study. The first lies in this study’s retrospective nature. Retrospective studies rely on chart notes from which important data may be lacking, potentially increasing bias incidence. Second, this study was conducted in a national referral centre for musculoskeletal infection and complex reconstruction, which introduces a significant selection bias. Patients referred to our institution often represent the most severe and complex cases, characterised by advanced infection, multiple prior surgical procedures, compromised soft tissues, and unfavourable host factors. Consequently, the proportion of patients requiring soft tissue reconstruction—particularly microsurgical free flaps—may be higher than that observed in non-referral settings. Third, four different surgeons carried out the study procedures, inevitably adding some variability of results. To balance this fact to some degree, all four surgeons were familiar with the protocol described and followed it faithfully. Other limitations to consider are the sample size and the follow-up period; even so, both of these factors were comparable or superior to those of previously published studies. Therefore, while our results demonstrate what can be achieved within a specialised setting, they should not be interpreted as evidence that similar outcomes can be expected universally. Rather, they support the concept that concentration of complex PJI care within experienced centres may be necessary to reproduce such results.

## 5. Conclusions

In chronic knee periprosthetic joint infection treated with a two-stage exchange protocol, the need for soft tissue reconstruction reflects increased clinical and biological complexity rather than an unfavourable prognostic factor. When embedded within a structured multidisciplinary orthoplastic approach, soft tissue reconstruction—including microsurgical free flaps—enables high rates of infection eradication, even in severely compromised cases. Differences between pedicled and free flap reconstruction primarily relate to defect extent and host factors, underscoring the importance of early referral and centralisation of complex PJI care in specialised centres.

## Figures and Tables

**Table 1:** Baseline demographic and clinical characteristics of N-STR and STR groups.

Demographic and Clinical Characteristics	N-STR Group (n = 78)	STR Group (n = 40)	*p* Value
Sex (Male/Female)	44.9%/55.1%	46.5%/53.5%	0.8462
Age, mean ± SD (years)	70.5 ± 9.27	68 ± 12.45	0.4206
Diabetes mellitus	28.2%	39.5%	0.2887
Presence of sinus tract	26.9%	44.8%	0.1022
Malignancy	14.1%	2.6%	0.1002
Smoking status	5.13%	13.16%	0.1509
Obesity	23.08%	15.79%	0.4667
Charlson Comorbidity Index, mean ± SD	4.13 ± 2.38	3.81 ± 2.40	0.5862
Inflammatory disease	6.41%	10.53%	0.4722
Cirrhosis	1.28%	0%	0.999
Anticoagulant therapy	10.26%	10.53%	0.999
Intra-articular pus	33.33%	31.58%	0.999
Suppressive antibiotic therapy	1.28%	2.70%	0.5419
Previous surgical procedures, mean ± SD	2.08 ± 1.32	3.03 ± 1.96	0.0057
ASA I	2.56%	5.56%	0.590
ASA II	39.75%	38.89%	0.999
ASA III	55.13%	52.78%	0.842
ASA IV	2.56%	2.78%	0.999
BMI	27.8 ± 4.51	26.7 ± 3.80	0.301

N-STR: No soft tissue reconstruction. STR: Soft tissue reconstruction. SD: Standard Deviation. BMI: Body Mass Index.

**Table 2:** Microbiological profile of N-STR and STR groups.

Microbiological Profile	N-STR Group (n = 78)	STR Group (n = 40)	*p* Value
*Methicillin-sensitive Staphylococcus aureus*	3.85%	12.5%	0.1903
*Enterococcus faecalis*	3.85%	12.5%	0.1903
*Enterobacter* spp.	1.28%	9.38%	0.0732
Other microorganisms	Low and comparable rates	Low and comparable rates	-
Polymicrobial infection	15, 68%	21, 42%	0.690

**Table 3:** Baseline demographic and clinical characteristics of GM flap and ALT flap reconstruction groups.

Demographic and Clinical Characteristics	GM Flap (n = 25)	ALT Free Flap (n = 15)	*p* Value
Sex (Male/Female)	40%/60%	53.3%/46.7%	0.512
Age, mean ± SD (years)	69.6 ± 13.55	65.6 ± 10.56	0.1224
Diabetes mellitus	60.87%	6.67%	**0.0017**
Presence of sinus tract	47.62%	37.50%	0.6968
Malignancy	0%	6.67%	0.3947
Smoking status	8.70%	20%	0.3649
Obesity	13.04%	20%	0.6632
Charlson Comorbidity Index, mean ± SD	3.68 ± 1.95	4 ± 2.94	0.6880
Inflammatory disease	0%	26.67%	**0.0185**
Immunodeficiency	0%	53.33%	0.0001
Anticoagulant therapy	17.39%	0%	0.1385
Intra-articular pus	8.33%	71.43%	**0.0095**
Suppressive antibiotic therapy	0%	7.143%	0.3784
Previous surgical procedures, mean ± SD	3.3 ± 1.65	2.67 ± 2.24	0.1335
Subsequent surgical procedures, mean ± SD	0.75 ± 1.55	2.5 ± 2.09	0.0022

GM: Medial Gastrocnemius Muscle Flap. ALT: Anterolateral Thigh Flap.

**Table 4:** Microbiological profile of GM and ALT flap reconstruction groups.

Microbiological Profile	GM Flap (n = 25)	ALT Free Flap (n = 15)	*p* Value
Positive cultures	32%	40%	0.74
Polymicrobial infection	25%	16.7%	0.999

**Table 5:** Postoperative complications of GM and ALT flap reconstruction groups.

Postoperative Complications	GM Flap (n = 25)	ALT Free (n = 15)	*p* Value
No complications	94.1%	46.7%	**0.0049**
Wound dehiscence	5.9%	6.7%	0.999
Partial flap necrosis	0%	26.7%	**0.038**
Total flap necrosis	0%	13.3%	0.212
Other complications	0%	6.7%	0.469

## Data Availability

The original contributions presented in this study are included in the article. Further inquiries can be directed to the corresponding author.

## Abbreviations

The following abbreviations are used in this manuscript:

PJI	Periprosthetic joint infection
EBJIS	European Bone and Joint Infection Society
N-STR	No soft tissue reconstruction
STR	Soft tissue reconstruction
ASA score	American Society of Anesthesiologists score
GM	Medial Gastrocnemius Muscle Flap
ALT	Anterolateral Thigh Flap
